# Effect of Nanosilver Gel, Chlorhexidine Gluconate, and Camphorated Phenol on *Enterococcus faecalis* Biofilm

**DOI:** 10.1155/2014/380278

**Published:** 2014-10-19

**Authors:** Dong Bo, Cecilia Marcellino Kayombo

**Affiliations:** Department of Conservative Dentistry and Endodontics, Unit I, Second Affiliated Hospital of Jiamusi University, No. 522 Hong Qi Road, Jiamusi, Heilongjiang 154002, China

## Abstract

*Aim*. To assess the effectiveness of nanosilver gel (NSG) in comparison to chlorhexidine gluconate (CHX) and camphorated phenol (CP) against* Enterococcus faecalis* (E.f) biofilm. *Methods and Materials*. Two tests were done, methyl thiazolyl tetrazolium (MTT) assay and confocal laser scanning microscopy (CLSM) analysis, to determine the effectiveness of NSG, CHX, and CP on E.f biofilm. Polystyrene microtiter 96- and 6-well plates were used for MTT and CLSM, respectively. Nanosilver gel was in three concentrations (0.05%, 0.1%, and 0.2%), chlorhexidine gluconate used was 2%, and camphorated phenol and normal saline were as control. Analysis was done using one-way ANOVA; the post hoc test was run for multiple comparisons. The level of statistical significance was set at *P* < 0.05. *Results*. One-way ANOVA showed significant differences among groups (0.05% NSG and CP, 0.1% NSG and CP, 0.2% NSG and CP, 0.1% NSG and 2% CHX, 0.2% and NSG and 2% CHX) (*P* < 0.001) and also showed significant difference between groups (*P* < 0.001), *f*-ratio 87.823. A post hoc Tukey's test revealed no significant difference between chlorhexidine gluconate and 0.05% nanosilver gel (*P* > 0.05). *Conclusions*. 0.1% and 0.2% nanosilver gel is more effective on *Enterococcus faecalis* biofilm as compared to chlorhexidine gluconate and camphorated phenol.

## 1. Introduction

Microorganisms and their by-products are considered to be the major cause of pulp and periradicular pathosis. Anaerobic bacteria especially black-pigmented Gram-negative species have been linked to the signs and symptoms of these diseases [1]. Facultative bacteria such as* Enterococcus faecalis* have also been isolated from infected root canal treatment [2].* E. faecalis* has been frequently found in root canal-treated teeth in prevalence values ranging from 30% to 90% of the cases. Root canal-treated teeth are about nine times more likely to harbor* E. faecalis* than cases of primary infections [3]. Its prevalence in such infections ranges from 24% to 77%. This finding can be explained by various survival and virulence factors possessed by* E. faecalis*, including its ability to compete with other microorganisms, invade dentinal tubules, and resist nutritional deprivation [4].

Various nanoparticles have gained popularity as antimicrobial agents as a result of their broad spectrum of activity and biocompatibility; recent studies have focused on using nanoparticulate materials to disinfect root canals. Nanosilver (NS) shows antibacterial effect; it also exhibits novel physicochemical and biological activities [5].

Chlorhexidine is a synthetic cationic bis-guanide that consists of two symmetric 4-chlorophenyl rings and two biguanide groups, connected by a central hexamethylene chain [6]. At higher concentration (2%), CHX is bactericidal as precipitation of the cytoplasmic contents occurs, which results in cell death [7]. Also canal dressing for 1 week with 2% CHX may provide residual antimicrobial activity against* E. faecalis* [8].

Camphorated phenol is among phenolic group of medicaments which have been applied either on a cotton wool pellet placed in the pulp chamber or on a paper point placed in the root canal, with the rationale being that the antimicrobial effect is delivered through vaporization of the medicament [9]. The antibacterial action of phenolic materials may not persist for prolonged periods of time. Hence, some bacteria may survive and have opportunity to multiply and persist in the root canal system [10].

The aim of this study was to compare the effectiveness of various concentrations of nanosilver gel with 2% chlorhexidine gluconate and camphorated phenol on* Enterococcus faecalis *biofilm using methyl thiazolyl tetrazolium (MTT) assay and confocal laser scanning microscopy (CLSM) analysis.

The hypothesis tested was that nanosilver gel, chlorhexidine gluconate, and camphorated phenol are equally effective on* Enterococcus faecalis* biofilm.

## 2. Materials and Methods

Two tests were done (MTT assay and CLSM analysis) to determine the effectiveness of nanosilver gel, chlorhexidine gluconate, and camphorated phenol on* E. faecalis* biofilm.

### 2.1. Bacteria Preparation

Pure strain of* E. faecalis* (ATCC 29212) from Nanjing Biotechnology Co. Ltd. was used, to create the bacterial inoculum; isolated colonies (24 hours) of pure cultures of E.f grown aerobically on brain heart infusion (BHI) agar plates were suspended in 5.0 mL BHI. The cell suspension was spectrophotometrically adjusted to match the turbidity equivalent to 0.5 McFarland standards.

### 2.2. MTT Assay

Polystyrene microtiter 96-well plates were used to evaluate the effect of medicaments on* E. faecalis* biofilm.

#### 2.2.1. Day 1

50 *μ*L of the 0.5 McFarland standards inoculum* E. faecalis* prepared was added to 1–7 columns and A–G rows and then 150 *μ*L of sterile BHI liquid medium was added to the microtiter plate where bacterial cells were seeded plus one column more (1–8 columns); the plate was then covered with the lid and sealed with parafilm and incubated at 37°C for 24 hours afterwards.

#### 2.2.2. Day 2

The biofilm was formed on the base of the plate after 24 hours; 50 *μ*L of the experimental medicaments was added to different columns to test their effectiveness. The plate was sealed and then incubated at 37°C for 24 hours. 

Medicaments were as follows:

1. normal saline (Shandong Qidu Pharmaceutical Co. Ltd.) as positive control;
2. 0.05%, 0.1%, and 0.2% nanosilver gel (Shenyang Dekang Medicine Technology Co. Ltd);
3. 2% chlorhexidine gluconate (Shantou Makat Hi-tech Co. Ltd);
4. camphorated phenol (Hubei Taichen Janrui Pharmaceutical Co. Ltd).

#### 2.2.3. Day 3

The medium was carefully aspirated; then each well was rinsed with sterile phosphate buffered solution (PBS) 2-3 times for about 5 minutes during each wash. 10 *μ*L of MTT solution was then added to the experimental wells, the plate was covered with aluminium foil to attain dark environment, and it was there after incubation at 37°C for 4–6 hours. After 4–6 hours the medium was aspirated and 150 *μ*L of dimethyl sulfoxide (DMSO) was added to each experimental well. The plate was kept in a microplate reader where it was shaken first for 10 minutes and then the absorbance value was measured at 630 nm wavelength.

### 2.3. CLSM Analysis

Polystyrene microtiter 6-well plates were used.

#### 2.3.1. Day 1

Sterile 22 × 22 coverslip was placed in a 6-well culture plate and left there for five minutes. 500 *μ*L of the 0.5 McFarland standards inoculum* E. faecalis* prepared was added on the surface of the coverslip and left there for 5 minutes. Then carefully 5 mls of sterile BHI liquid medium was added along the side walls of the plate; the plate was sealed with the parafilm and then incubated at 37°C for 24 hours for formation of* E. faecalis* biofilm on glass coverslips.

#### 2.3.2. Day 2

200 *μ*L of the experimental medicaments was added; the plates were sealed and then incubated at 37°C for 24 hours. 

#### 2.3.3. Day 3

The culture medium and medicine were carefully aspirated without touching the coverslip. The cells were then washed twice with sterile PBS solution for 2 minutes each time. 300 *μ*L of the prepared AO/EB dye solution was added, and the plates were left in the dark for 15 minutes. Then the specimens were observed under CLSM with absorbance wavelength of 543 nm for AO (green) and 488 nm for EB (red) dyes under 200 magnification.

## 3. Results

### 3.1. MTT Assay

At the absorbance of 630 nm the results are shown in Table 1.

Mean and standard deviation values for each experimental group and control groups were calculated using SPSS 20.0 software and the percentage inhibition for each group of experimental drug was calculated using the following formula below and results are shown in Table 2:

(1) %  inhibition =Control  OD  value−Experimental  group  OD  valueControl  OD  value − Blank  control  OD  value  ×100%.

Statistical analysis using one-way ANOVA showed significant differences among groups (0.05% NSG and CP, 0.1% NSG and CP, 0.2% NSG and CP, 0.1% NSG and 2% CHX, and 0.2% NSG and 2% CHX) (*P* < 0.001) and also showed significant difference between groups (*P* < 0.001), *f*-ratio 87.823. A post hoc Tukey's test revealed no significant difference between chlorhexidine gluconate and 0.05% nanosilver gel (*P* > 0.05).

### 3.2. CLSM Analysis

**Figure 1:**
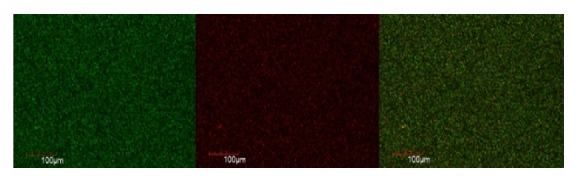
Positive control group.

**Figure 2 fig2:**
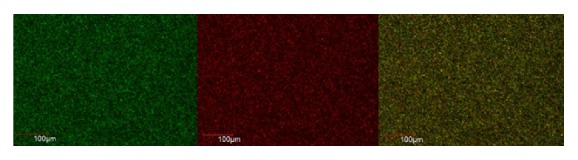
Camphorated phenol.

**Figure 3 fig3:**
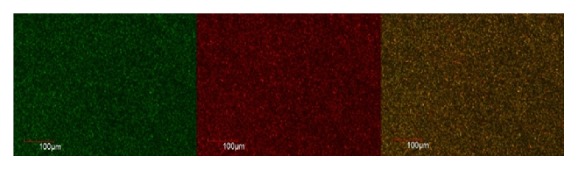
2% chlorhexidine gluconate.

**Figure 4 fig4:**
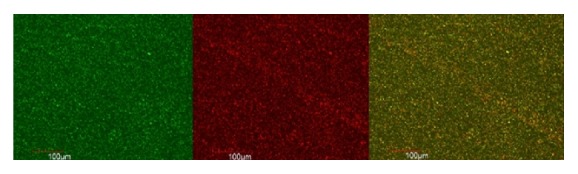
0.05% nanosilver gel.

**Figure 5 fig5:**
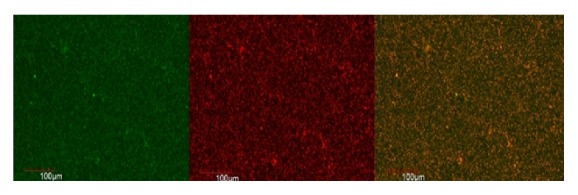
0.1% nanosilver gel.

**Figure 6:**
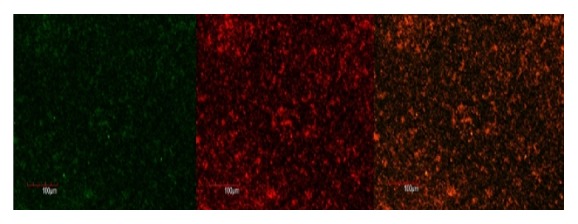
0.2% nanosilver gel.

The live and dead cells of E.f in the biofilm formed on the coverslip were observed according to the uptake of green and red/orange dye by the bacterial cells. Figures 1, 2, 3, 4, 5, and 6 show CLSM images of bacteria biofilm treated with different groups of medicaments. The colour of the biofilm changes from green to yellowish red, nanosilver gel being more yellowish red (in all 3 concentrations) compared to 2% chlorhexidine gluconate and camphorated phenol and more green for the control group indicating that almost all the cells are alive (Figures 1 [Fig fig2] [Fig fig3] [Fig fig4] [Fig fig5]–6).

## 4. Discussion

The term biofilm was introduced to designate the thin layered condensations of microbes that may occur in various surface structures in nature. Free floating bacteria existing in an aqueous environment, the so-called planktonic form of microorganisms, are prerequisite for biofilm formation [11]. Biofilms may thus become established on any organic or inorganic surface substrate where planktonic microorganisms prevail in a water based solution. In dental context, a well-known and extensively studied biofilm structure is dental plaque. Here, bacteria free in saliva (planktonic organisms) serve as primary source of organisms for the organization of this specific biofilm [11]. In endodontics, the biofilm concept was initially discussed mainly within the framework of bacteria on the root tips of the teeth with necrotic and infected pulps and infected root canals. Such bacteria aggregations have been thought to be the cause of therapy-resistant apical periodontitis [12]. Although not described in as much detail, bacterial condensations (biofilm) on the wall of infected root canals have been observed.

Antimicrobial agents have often been developed and optimized for their activity against fast growing, dispersed populations containing a single microorganism. However, microbial communities grown in biofilms are remarkably difficult to eradicate with antimicrobial agents and microorganisms in mature biofilms can be notoriously resistant for the reasons that have yet to be adequately explained [11]. There are reports showing that microorganisms grown in biofilms could be twofold to 1000-fold more resistant than the corresponding planktonic form of the same organisms [13].* E. faecalis* has a strong tendency to form biofilm in the root canals and hence has a tendency of having antibiotic resistance to conventional therapy and is also proved to be resistant to the most widely used medicaments to disinfect the canals.

Therefore, an endodontic irrigant/medicament should ideally exhibit powerful antimicrobial activity, disinfect the root canal space, and have no cytotoxic effect on periradicular tissues, among various other properties required. Hence, an equally effective and safe irrigant/medicament is desirable [14].

Confocal laser scanning microscope (CLSM) has provided the ability to examine biofilms in situ without the limitations encountered with the SEM, albeit at lower magnifications. CLSM is now being used to determine the true architecture of plaque and the location of selected bacteria within the biofilm. The use of CLSM requires that the organisms in the biofilms be stained with fluorescent stains. These stains are designed to emit light at specific wavelengths and can be used to probe specific cellular functions. Using a suite of such stains allows the biofilm researcher to quantify all the cells and determine which ones are viable [15].

The null hypothesis of the present study has been rejected. Nanosilver gel was found to be more effective than chlorhexidine gluconate and camphor phenol against* E. faecalis* biofilm. The results of this study demonstrated antibacterial activity against bacterial strains by all the intracanal medicaments tested. The bacterial strains chosen for this study belong to bacterial species that are clinically relevant, since they are part of the endodontic pathogens and are also new emerging pathogens causing infections in other clinical fields [16–18]. The microorganism in the current study is a common isolated pathogen in both primary and secondary endodontic infections.

The silver nanoparticles showed efficient antimicrobial property compared to other medicaments due to their extremely large surface area, which provides better contact with microorganisms. The nanoparticles get attached to the cell membrane and also penetrate inside the bacteria. The bacterial membrane contains sulphur-containing proteins and the silver nanoparticles interact with these proteins in the cell as well as with the phosphorus containing compounds like DNA. When silver nanoparticles enter the bacterial cell, it forms a low molecular weight region in the centre of the bacteria to which the bacteria conglomerate, thus protecting the DNA from the silver ions. The nanoparticles preferably attack the respiratory chain, cell division finally leading to cell death. The nanoparticles release silver ions in the bacterial cells, which enhance their bactericidal activity [19–22].

The biologic effects of silver are believed to be closely related to silver ion [23]. Studies reported that silver nanoparticles are cytotoxic to different cell lines. This toxicity is only partially recognized [23]. Results showed that silver nanoparticles are cytotoxic in the case of exposure at high concentrations [24]. There are contradictory studies on silver nanoparticles and ion cytotoxicity from laboratories around the world. Silver is known to have a lethal effect on bacteria, but the same property that makes it antibacterial may render it toxic to human cells. Concentrations of silver that are lethal for bacteria are also lethal for both keratinocytes and fibroblasts [25]. In vitro studies have demonstrated that nanosilver has effects on reproduction and development and has an effect on DNA among others. In contrast, it was found out that adding 1.0% silver nanoparticles (5–50 nm) to bone cement, a dose at which bactericidal activity was seen, did not result in (additional) cytotoxicity towards mouse fibroblasts (L929) or on growth of human osteoblast cell line (hFOB 1.19) [26].

Results of the current study indicate a dose dependent antimicrobial activity of nanosilver especially when compared with chlorhexidine gluconate; nanosilver gel showed the ability to inhibit* Enterococcus faecalis* biofilm formation at different concentrations, even at much lower concentration than chlorhexidine gluconate.

The current study shows that camphorated phenol has lower inhibition effect compared to nanosilver gel and 2% chlorhexidine gluconate which coincides with the finding when camphorated phenol was compared to calcium hydroxide [27].

It was found out that CHX was less effective in eliminating E.f biofilm [28] which concurs with the present study. On the contrary, it was found out that CHX was more effective in eliminating E.f biofilm compared to other medicaments [29].

## 5. Conclusion

According to the results of the current study, at a very low concentration nanosilver gel can be used to reduce bacteria load especially the most resistant* E. faecalis* as intracanal medicament. More studies using animal models and clinical studies are to be done to get a better understanding of the effects of nanoparticles on periodontal tissues; also more data on the cytotoxicity of silver nanoparticles are needed on appropriate model organisms.

## Figures and Tables

**Table 1:** Optical density values for experimental medicaments.

Negative control	Positive control	0.05% NSG	0.1% NSG	0.2% NSG	2% CHX	CP	Blank control
(nm)	(nm)	(nm)	(nm)	(nm)	(nm)	(nm)	(nm)
1.117	1.242	0.370	0.264	0.257	0.452	0.764	0.029
1.208	1.108	0.195	0.264	0.160	0.471	0.750	0.027
1.244	1.205	0.231	0.253	0.094	0.478	0.845	0.033
1.153	1.227	0.266	0.264	0.155	0.458	0.863	0.030
1.269	1.263	0.677	0.255	0.257	0.462	0.720	0.033
1.268	1.244	0.812	0.253	0.253	0.450	0.678	0.030
1.246	1.237	0.454	0.260	0.152	0.465	0.641	0.030

Note that negative control contained BHI medium and bacteria cells and positive control contained BHI medium, bacteria cells, and normal saline, while blank control contained BHI medium only.

**Table 2:** Percentage inhibition for experimental drugs.

Drug	Mean OD ± SD values	% inhibition
NS (positive control)	1.2180 ± 0.0516	4.7
0.05% NSG	0.4293 ± 0.2355	67
0.1% NSG	0.2590 ± 0.0052	78
0.2% NSG	0.1897 ± 0.0655	86
CHX	0.4623 ± 0.0100	63
CP	0.7516 ± 0.0816	39
